# Camping Duties of the Territorial Regimental Surgeon

**Published:** 1909-07-24

**Authors:** 


### The Royal Army Medical Corps Section.

### CAMPING DUTIES OF THE TERRITORIAL REGIMENTAL SURGEON.

In connection with the annual training camp of his unit, a number of special duties fall to the lot of the regimental medical officer, and with these he should make himself fully acquainted. One of the most important is the preparation and despatch of the preliminary indent for the medical equipment required while in camp. The date for sending in this requisition is usually announced in the command orders, as it has to be sent to the regular principal medical officer of the command, but through the administrative medical officer of the division.

The amount of medical equipment is laid down in Appendix XI. of the regulations (T. F.), and for a regimental unit of 500 men or more consists of one medical companion and water-bottle, and of two surgical haversacks and water-bottles. It should be applied for on Army Form I 1209, which should be signed by the medical officer. On each requisition there should be stated the following details: (1) The name of the unit for which the application is made; (2) where the unit is to be encamped; (3) the place where the stores are to be sent to; (4) the date and time of day at which they should arrive; and (5) to whom they should be delivered. Unless all the above particulars are given, the district loan equipment department cannot carry out the order correctly. Experience shows that regimental medical officers are not sufficiently careful in giving all the above details, thereby necessitating the return of the army form for its proper completion or correction, and thus causing both delay and extra work.

A medical officer may receive his camp equipment before leaving home or on arrival in camp, but wherever it is received he should at once check it with the lists supplied, so that any deficiencies may be at once noted and responsibility for them disclaimed, otherwise he will be held liable for them when the equipment is handed over at the termination of the camp. Another very important matter to note on arrival is the condition of the camp as to cleanliness and sanitary accessories, as was pointed out in the R.A.M.C. article of June 12 in this journal.

The daily routine duties of the regimental medical officer when in camp are varied and important, and call for a certain amount of system and method for their proper discharge. Fortunately he is not required to attend parades except in a professional capacity, exemption being specially given by paragraph 1096 of the King's Regulations. By another paragraph, 1097, he is also exempted from being present at target practice, it being sufficient to communicate with the commanding officer of the unit, saying where he can be found if required while the shooting is going on. The care of the sick of his unit is always a constant thought to the regimental medical officer, for the means at his disposal for dealing with them are limited. All the hospital equipment he has at his command are two small bell tents, one of which can serve as an inspection tent or office, and in the other as an observation tent for any special case. He must see all men reporting sick and must visit them daily, as he is responsible for them from the moment they come under his care.

The procedure to be followed varies. Cases of temporary indisposition are usually treated in their own quarters (tents), but if the medical officer thinks it necessary, a case may be transferred to the observation tent. More serious cases that will probably be unfit for duty for some days are sent to an adjacent military hospital, or, in the absence of the latter, to any neighbouring civil hospital by previous arrangement with the P.M.O. of the command. On the other hand, should it be apparent that the patient is not likely to be fit for duty during the camping period, it is better to have him discharged and his return home arranged for. A medical officer should exercise special vigilance for cases of infectious disease in the early days of the camp. When it is remembered that the men of the unit come straight from their homes to camp, the chances of infection from so many different sources are considerable, and great watchfulness is necessary.

Whenever a man is found to be suffering from infectious disease, whether on joining the camp or subsequently, he must be detained and isolated, and notice at once sent to the medical officer of health for the district, with a view to the man's transfer to the local hospital for infectious diseases, as laid down in paragraph 310 of the Regulations (T.F.). The field days that are held during camp training furnish opportunities for the care of the sick while on the line of march, and the medical officer must remember that he is responsible not only for the professional treatment of the men admitted to the ambulance wagons, but also for their discipline, as laid down in paragraph 1908 of the King's Regulations.

Of greater importance even than the care of the sick is the preservation of health amongst the men of a regiment. To prevent disease must be the medical officer's constant aim, and it can only be attained by attention to details. The number occupying a tent, its ventilation, its cleanliness, are matters to be inquired into. Tents are best aired by tying up the "flies," and it is a good plan to sleep with them so. Daily removal into the open of the bedding and kits of the men is a most necessary procedure, if the weather allows, as the cleansing value of fresh air and sunshine cannot be over-estimated.

Scrupulous cleanliness in the tents, especially in connection with meals, and the avoidance of the dangerous and objectionable habit of spitting on the floor of a tent are two most important points to emphasise. A medical officer should satisfy himself also that the water supply does not suffer any pollution during camp; that all waste water, especially greasy water, is suitably disposed of; that the excreta are properly dealt with, and that all rubbish, as well as the wastage from lines and tents, is burnt in an incinerator. Especially should the cook-houses where the food for the officers and the men is prepared be visited daily, and a very high standard of cleanliness amongst the cooks, waiters, and officers' servants should be insisted on in this department.

The hours of meals and the standard of cooking which is provided are matters that should not be overlooked, while on field days the arrangements for feeding the men should have special consideration, as they may not get dinner until very late in the day. In connection with field days every medical officer should remember that the fitness of his regiment depends on its marching powers, and that this in turn hangs very much on the state of the men's feet. Instruction on this subject, especially as to the treatment of blistered feet, should be given, and foot parades for the inspection of the men's feet should be held, while minor points, as the condition of the boots and socks, should not be beneath notice.

At the close of a regimental camp the medical officer will see that the medical equipment is properly packed and returned to the district loan equipment store of the command, but he must first check it, and note all the deficiencies on the list which is supplied with each article. Having carefully recorded all losses on Army Form I 1230, it should be signed and dated by the medical officer and forwarded to the A.M.O. of the division. This, however, does not completely finish the camp duties of the medical officer. He has still to forward to the administrative medical officer of the division a report on the sanitary arrangements, the state of health and general working of the camp. This should be done at the end of the training, and should be an official document on the lines laid down in the King's Regulations.

In view of the necessity for this report a medical officer should make notes while in camp to help him in compiling his final copy, which should be sent in as soon as possible while matters are fresh in his memory, for the work of one of these temporary regimental camps is of a heterogeneous and varied nature, and necessitates daily memoranda being made if accuracy is to be attained. In conclusion, it may be noted that the remarks in this article are confined to duties in a regimental camp. They differ somewhat from those in a brigade camp, which are best considered separately.

